# Pulmonary Nocardia farcinica Infection in a Non-immunocompromised Patient With Fibrotic Nonspecific Interstitial Pneumonia (NSIP)

**DOI:** 10.7759/cureus.89074

**Published:** 2025-07-30

**Authors:** Narendhar V, Dhanasekar Thangaswamy

**Affiliations:** 1 Department of Pulmonology, Sri Ramachandra Institute of Higher Education and Research, Chennai, IND

**Keywords:** aluminium exposure, corticosteroids, interstitial lung disease, nocardia farcinica, opportunistic infection, pulmonary nocardiosis

## Abstract

Pulmonary nocardiosis is a rare and often overlooked infection, particularly in individuals without classical immunosuppression. This report describes an unusual presentation of *Nocardia farcinica* pulmonary infection in a patient with fibrotic interstitial lung disease and a significant history of occupational exposure to aluminum fumes. A 56-year-old male patient presented with progressive respiratory symptoms and hemoptysis, ultimately diagnosed through microbiological culture. Clinical improvement followed targeted antibiotic therapy. This case highlights the importance of considering atypical infections in patients with chronic lung conditions and environmental exposures.

## Introduction

Nocardia is a genus of filamentous, aerobic Gram-positive bacteria that are widely distributed in soil and decaying organic matter. Although primarily saprophytic, several species within this genus are pathogenic to humans. Since Edmond Nocard first described the organism in 1888, over 80 species have been identified, more than 30 of which are known to cause disease in humans. Notable among these are the Nocardia asteroides complex, N. brasiliensis, N. farcinica, and the emerging N. cyriacigeorgica [1,2]. The taxonomic classification of Nocardia has undergone substantial revision in recent decades, facilitated by molecular diagnostic tools such as 16S ribosomal RNA (rRNA) sequencing and multilocus sequence analysis (MLSA). These methods have significantly enhanced the precision of species identification, particularly in differentiating morphologically similar organisms [1,3,4].

Clinically, nocardiosis manifests most commonly as pulmonary or cutaneous infections, though dissemination to the central nervous system and other organs is well-documented, especially in immunocompromised individuals. This includes patients with HIV/AIDS, malignancies, chronic lung diseases, or those receiving immunosuppressive therapies such as corticosteroids or chemotherapy [2,5]. Diagnostic challenges are common, as Nocardia infections often mimic tuberculosis, fungal infections, or neoplastic processes in both clinical and radiologic presentations [2,6]. The organism's slow growth and variable antimicrobial resistance further complicate timely diagnosis and management. Recent epidemiological trends suggest an increasing incidence of Nocardia infections, particularly in individuals with underlying pulmonary conditions like bronchiectasis. This rise is likely attributed to heightened clinical vigilance and improved diagnostic capabilities [5,7].

Emerging species such as N. cyriacigeorgica have been increasingly reported in the United States [8], while atypical presentations such as brain abscesses and severe pulmonary involvement continue to be described in immunocompromised populations [9,10]. The present case highlights a rare instance of pulmonary Nocardia farcinica infection in a patient with fibrotic nonspecific interstitial pneumonia (NSIP) and chronic occupational exposure to aluminum fumes, contributing to the existing literature on atypical presentations of nocardiosis.

## Case presentation

A 56-year-old male with a known history of type 2 diabetes mellitus and fibrotic NSIP presented in July 2024 with a two-year history of productive cough and progressive dyspnea, which had advanced from modified Medical Research Council (mMRC) grade 2 to grade 4. The patient also reported 10 episodes of hemoptysis, each approximately 5 mL in volume, within a 24-hour period. Notably, he had been employed in the aluminum industry for over two decades, where he was routinely exposed to metal fumes without adequate protective equipment or ventilation. He denied any history of smoking or pet exposure.

One month prior to presentation, he had been diagnosed with hypersensitivity pneumonitis at another healthcare facility and was prescribed oral prednisolone at a dose of 20 mg per day for 20 days. Upon admission, he was hypoxemic with a room air oxygen saturation of 70%, tachycardic at 110 beats per minute, and hypotensive with a blood pressure of 100/60 mmHg. Arterial blood gas on room air showed a pH of 7.47, PaO₂ of 52 mmHg, PaCO₂ of 30 mmHg, HCO₃⁻ of 22 mEq/L, and SaO₂ of 70%, consistent with type 1 respiratory failure and CO₂ washout due to hyperventilation. Physical examination revealed grade 3 digital clubbing and bilateral basal crepitations on auscultation.

**Figure 1:**
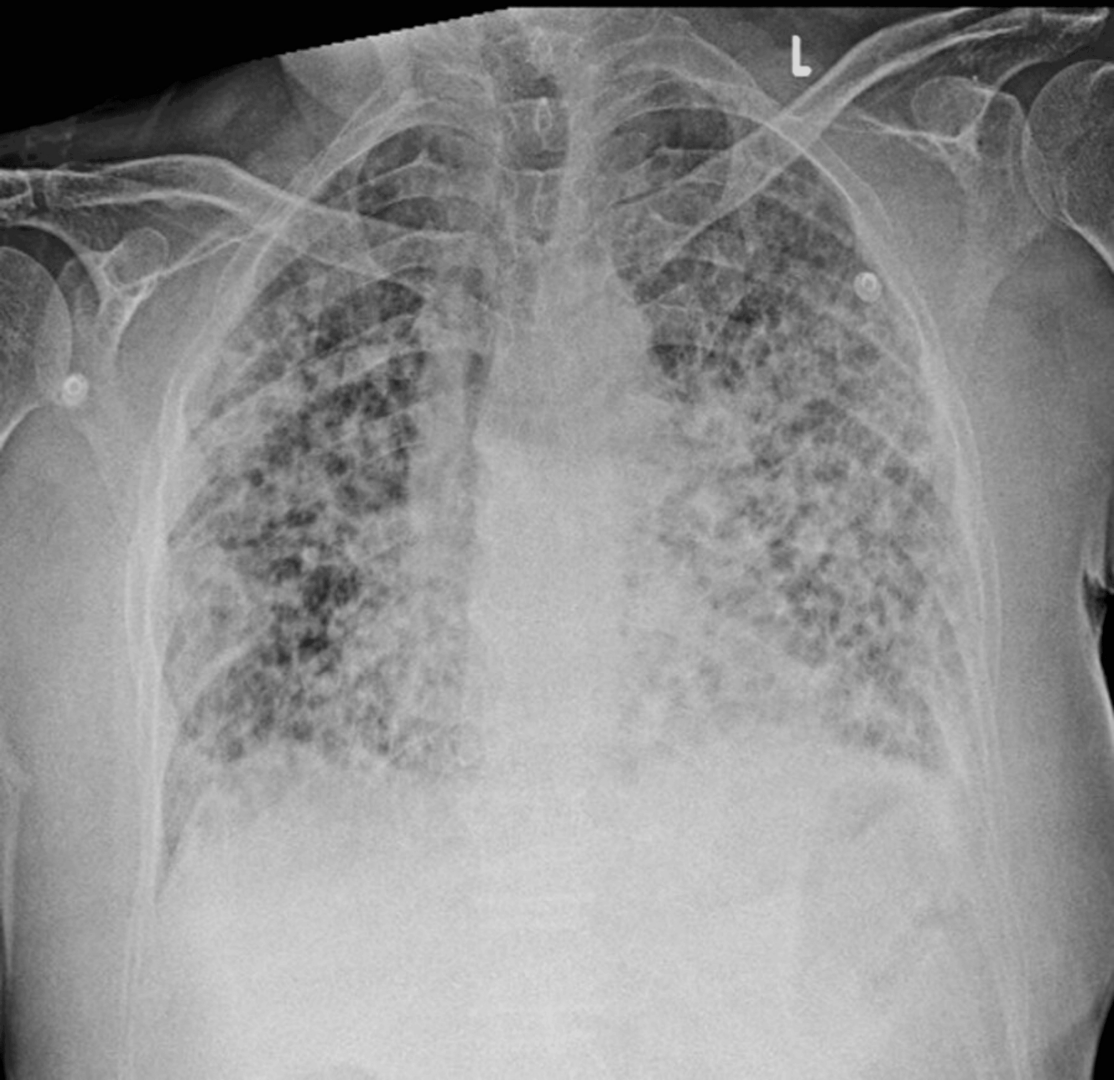
Chest X-ray showing bilateral upper, mid and lower zones reticular opacities

**Figure 2:**
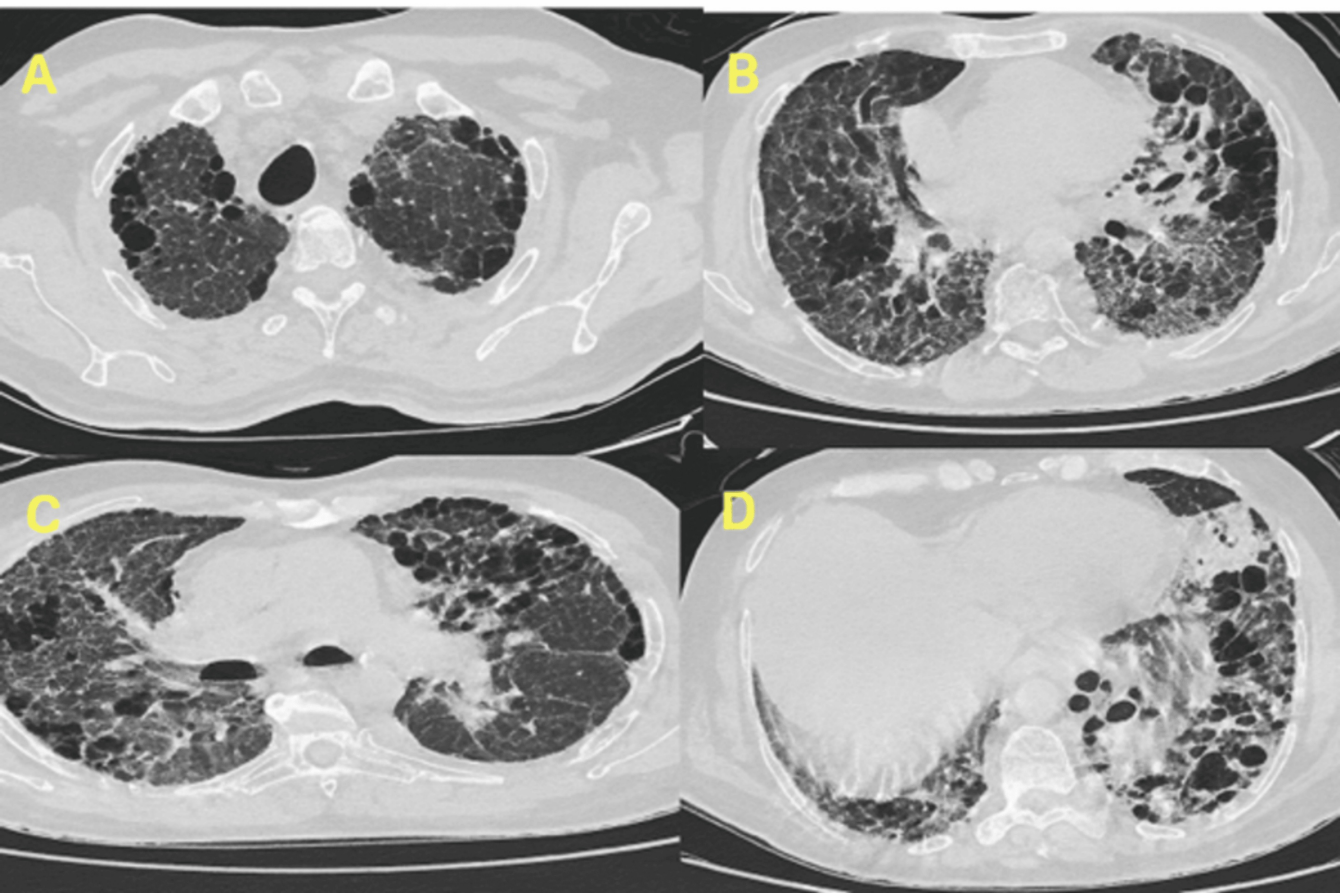
High-resolution CT thorax demonstrating advanced fibrotic interstitial lung disease: Subpleural macrocystic and microcystic honeycombing involving bilateral lung fields, with an apicobasal gradient. Diffuse mild ground-glass opacities with extensive admixed inter- and intralobular septal thickening, predominantly affecting the bilateral lower lobes. Fibrosis with traction bronchiectasis and architectural distortion, most marked in the lower lobes. A. Apical section: Extensive subpleural and peribronchovascular clustered cystic airspaces consistent with honeycombing, predominantly involving the bilateral upper lobes. B. Middle lung zones: Marked architectural distortion, traction bronchiectasis, and reticular opacities with continued honeycombing. C. Lower lung fields: Diffuse reticulation, traction bronchiectasis, and confluent cystic changes with basal predominance. D. Basilar section: Severe fibrotic changes, including extensive subpleural honeycombing with peripheral and lower lobe predominance, consistent with a usual interstitial pneumonia (UIP) pattern. These imaging findings correlate with advanced fibrotic interstitial lung disease, likely definitive UIP pattern.

High-flow nasal cannula (HFNC) oxygen therapy was initiated but subsequently escalated to non-invasive ventilation due to increased respiratory effort. The chest X-ray (Figure 1) showed bilateral upper zone, midzone and lower zone reticular opacities. High-resolution computed tomography (HRCT) (Figure 2 )of the chest demonstrated extensive subpleural honeycombing, diffuse ground-glass opacities, inter- and intralobular septal thickening, traction bronchiectasis, and architectural distortion consistent with a combined NSIP and usual interstitial pneumonia (UIP) pattern. A mild left-sided pleural effusion was also observed. Initial laboratory investigations were done and are elaborated in Table 1. 

**Table 1:** Quantitative and Qualitative Laboratory Investigations at Admission Table 1 summarizes both quantitative and qualitative investigations at admission, with reference values and interpretations. Significant findings included elevated inflammatory markers (CRP, procalcitonin), confirmed Influenza A, and a positive sputum culture for Nocardia species. ACE: angiotensin-converting enzyme, AFB: acid-fast bacillus, ANA: antinuclear antibodies, IF: immunofluorescence, ANCA: anti-neutrophil cytoplasmic antibody, ELISA: enzyme-linked immunosorbent assay, CCP: cyclic citrullinated peptide

Test/Parameter	Result	Reference Range / Interpretation	Interpretation
Hematology & Inflammatory Markers			
Hemoglobin	13.7 g/dL	13.0–17.0 g/dL	Normal
Total Leukocyte Count	9810 /mm³	4000–11,000 /mm³	Normal
Platelet Count	1.84 × 10⁵/μL	1.5–4.0 × 10⁵/μL	Normal
C-Reactive Protein (CRP)	12.7 mg/L	<5 mg/L	Elevated
Procalcitonin	4.47 ng/mL	<0.15 ng/mL	Markedly Elevated
Biochemistry			
Serum Creatinine	Normal	0.6–1.3 mg/dL	Within Normal Limits
Serum Electrolytes	Normal	NA⁺: 135–145 mmol/L etc.	Within Normal Limits
Calcium	7.5 mg/dL	8.5–10.5 mg/dL	Decreased
Serum ACE Levels	Normal	8–52 U/L	Normal
Autoimmune and Infectious Workup			
Influenza A (Respiratory PCR)	Positive	—	Positive
Sputum AFB / GeneXpert	Negative	—	Negative
Sputum Nocardia	Positive Grew* Nocardia farcinica*	—	Positive
ANA by IF	Negative	Negative	Negative
Myositis Profile	Negative	Negative	Negative
ANCA by ELISA	Negative	Negative	Negative
Rheumatoid Factor (RA)	Negative	Negative	Negative
Anti-CCP	Negative	Negative	Negative
Hypersensitivity Pneumonitis Panel	Negative	Negative	Negative

Empirical treatment was initiated with intravenous piperacillin-tazobactam, oral azithromycin, nebulized bronchodilators, and systemic corticosteroids. However, due to lack of clinical improvement, antibiotics were escalated to intravenous meropenem.

Subsequent sputum cultures grew Nocardia farcinica, which was sensitive to amikacin and cotrimoxazole. These agents were promptly added to the regimen. The patient received intravenous meropenem for 14 days, followed by amikacin plus oral cotrimoxazole for three weeks. Upon discharge, he was advised to continue oral cotrimoxazole for an additional three months, completing a total treatment duration of approximately six months.

Notably, respiratory PCR at admission was positive for Influenza A. This viral co-infection likely contributed to pulmonary epithelial injury, creating a susceptible environment for secondary bacterial invasion and worsening the clinical course.

Despite the absence of direct microbiological evidence of invasive fungal infection, voriconazole was initiated based on elevated serum β-D-glucan and galactomannan levels. The decision was guided by the patient’s immunocompromised state (due to corticosteroid use and underlying diabetes mellitus) and the potentially high mortality associated with undiagnosed fungal co-infections. The benefit-risk assessment favored early initiation of antifungal therapy in this context.

The patient gradually improved clinically, was successfully weaned to a Venturi mask, and was ultimately discharged in a stable condition with home long-term oxygen therapy (LTOT) on oral cotrimoxazole and advised close follow-up with repeat imaging and laboratory monitoring. However, he was lost to follow-up after discharge, and no further clinical information could be obtained regarding his long-term outcomes or treatment adherence.

## Discussion

Pulmonary nocardiosis remains a formidable diagnostic and therapeutic challenge, particularly in patients with pre-existing structural lung disease, even when systemic immunosuppression is not evident. This case highlights *Nocardia farcinica* infection in a patient with fibrotic NSIP and long-term occupational exposure to aluminum fumes. This unique combination likely contributed to localized pulmonary immune dysfunction.

Chronic lung disease is a well-established risk factor for nocardial infections. Woodworth et al. reported a higher incidence of Nocardia among patients with bronchiectasis at a tertiary care center. This underscores the vulnerability of structurally abnormal lungs to opportunistic pathogens [5]. Similarly, Baio et al. observed that many nocardiosis cases in Brazil occurred in patients without overt immunosuppression but with chronic pulmonary conditions [7]. Our findings are consistent with these observations. Like bronchiectasis, fibrotic NSIP may create a permissive environment for Nocardia colonization and infection.

Corticosteroid use, even for short durations, is another known predisposing factor. Clark and Reid emphasized the risk of nocardiosis in solid organ transplant recipients receiving immunosuppressants. However, they also noted that similar infections can occur in non-transplant patients treated with steroids [2]. In our patient, corticosteroid therapy likely induced localized immunosuppression, compounding the vulnerability from structural lung disease.

Radiological differentiation of nocardiosis from other pulmonary infections or malignancies is often difficult. Schlaberg et al. and Muricy et al. have shown that Nocardia can mimic tuberculosis and fungal infections on imaging. Common overlapping features include consolidation, nodules, and cavitary lesions [6,8]. In our case, the background of fibrotic NSIP further obscured these features. This made microbiological confirmation critical to diagnosis.

Molecular diagnostics have greatly enhanced the ability to identify Nocardia species. Conville et al. and McTaggart et al. demonstrated how 16S rRNA sequencing and multi-locus sequence analysis can improve species-level identification and guide appropriate treatment [1,3]. In our case, accurate identification of N. farcinica was crucial, given its known virulence and resistance profile.

Nocardia farcinica is particularly notorious for multidrug resistance and high pathogenicity. Traxler et al. described N. farcinica as one of the more clinically significant and resistant Nocardia species [4]. While cotrimoxazole remains the first-line agent, severe or resistant infections often require combination regimens. Shen et al. reported successful treatment of N. farcinica with linezolid, although they cautioned about potential hematologic side effects [10]. In our patient, antibiotic selection was tailored based on susceptibility results. Linezolid was considered but reserved due to safety concerns.

Dissemination, especially to the brain, is a well-recognized complication of nocardiosis. Chakrabarti et al. described a nocardial brain abscess in a diabetic patient, highlighting the organism’s neurotropic potential [9]. Although our patient had no neurological symptoms, his risk factors of corticosteroid use, diabetes, and fibrotic lung disease necessitated close monitoring for possible extrapulmonary spread.

## Conclusions

This case underscores the importance of considering Nocardia farcinica as a potential pathogen in patients with chronic structural lung disease, particularly those receiving immunosuppressive therapy or exposed to environmental lung irritants. Given the non-specific clinical and radiological manifestations, a high index of suspicion is required. Prompt microbiological diagnosis and targeted antimicrobial therapy can lead to favorable outcomes, even in complex clinical scenarios. Continued awareness and investigation are essential to improve the recognition and management of nocardiosis in atypical patient populations.
